# Health workers’ knowledge on future vascular disease risk in women with pre-eclampsia in south western Nigeria

**DOI:** 10.1186/s13104-015-1553-6

**Published:** 2015-10-16

**Authors:** D. A. Adekanle, A. S. Adeyemi, S. A. Olowookere, C. A. Akinleye

**Affiliations:** Department of Obstetrics and Gynaecology, College of Health Sciences, Ladoke Akintola University of Technology, Osogbo, Nigeria; Department of Community Health, College of Health Sciences, Obafemi Awolowo University, Ile-Ife, Nigeria; Department of Community Medicine, LAUTECH Teaching Hospital, Osogbo, Nigeria

**Keywords:** Health workers, Pre-eclampsia, Cardiovascular risk, Counseling

## Abstract

**Background:**

Pre-eclampsia progressing to eclampsia is one of the major causes of maternal death in Nigeria. Since there is long term association of pre-eclampsia with cardiovascular disease, cerebrovascular disease, renal disease, short life expectancy and mortality, it is essential to obtain obstetric history for better counseling and long term monitoring. The study assessed the knowledge of health workers about the association of pre-eclampsia with future cardiovascular disease and offering any risk-reduction counseling to women with pre-eclampsia.

**Methods:**

During a training workshop, a validated questionnaire on the association between pre-eclampsia and cardiovascular risk was distributed among health care workers working at the infant welfare and family planning clinics in Osun State. Data were analysed using descriptive and inferential statistics.

**Results:**

One hundred and forty-six out of 150 health workers approached participated in the study (response rate 97.3 %). Mean age of respondents was 35.6 ± 9.1 years. Median age of practice was 7 years, ranging from 1–40 years. They were medical doctors (60.3 %), community health workers (26.7 %) and nurses/midwives (13.0 %). Most participants had good knowledge on future cardiovascular risk of pre-eclampsia. The medical doctors had better knowledge compared to nurses/midwives and community health workers (78.4 vs. 57.9 vs. 53.8 %; p < 0.05). Below half (45.9 %) offered risk-reduction counseling.

**Conclusion:**

Knowledge of the cardiovascular risk factors was lower among the nurses/midwives and community health workers. Risk reduction counseling was quite low across all the health workers. There is need for continuous medical education and possible review of the training curriculum of the lower cadres of health workers.

**Electronic supplementary material:**

The online version of this article (doi:10.1186/s13104-015-1553-6) contains supplementary material, which is available to authorized users.

## Background

Pre-eclampsia is accountable for the world’s large maternal mortality rates, 60,000 per year worldwide [1]. The acute complications such as eclampsia, cardiovascular disease and other organ damage contribute to 75 % fatalities [2]. Pre-eclampsia progressing to eclampsia is one of the major causes of maternal death in Nigeria [3]. Recent studies have shown that pre-eclampsia has long-term associations with cardiovascular disease, cerebrovascular disease, renal disease, short life expectancy and mortality [4–6]. Pre-eclampsia still remains a disease of theory as the aetiology is not known, with only associated risk factors [7]. Emerging data from Hospital studies in Nigeria showed that cardiovascular disease or its complications is the commonest non-communicable disease with both men and women equally affected [8]. Large cohort studies have shown that pre-eclampsia is associated with higher risk of cardiovascular disease compared with studies without history of pre-eclampsia [5–7, 9, 10]. Various studies have supported the evidence that pre-eclampsia and other hypertensive disorders in pregnancy are associated with cardiovascular risk factors later in life [4, 11]. In the light of the facts highlighted, obstetric history forms an important risk factor for future cardiovascular disease. Some reports have suggested that women with history of pre-eclampsia should be counseled for future cardiovascular disease risk and be monitored closely for cardiovascular disease modifiable risk factors [11, 12]. The objective of this study was to assess the knowledge of health workers about the association of pre-eclampsia with future cardiovascular disease and offering any risk-reduction counseling to women with pre-eclampsia.

## Methods

### Study setting

Osun State was created from old Oyo State in 1991. It has 30 Local Government Areas with several maternity centres, Comprehensive Health Centres, and State Hospitals and two Teaching Hospitals: Ladoke Akintola University of Technology (LAUTECH) Teaching Hospital, Osogbo and Obafemi Awolowo University Teaching Hospital Complex, Ile-Ife, Nigeria.

This study was conducted in April of 2014 during a workshop organized by the Department of Obstetrics and Gynaecology, LAUTECH Teaching Hospital, Osogbo in conjunction with Osun State Ministry of Health and Osun State Local Government Service Commission. All medical doctors, nurses/midwives and community health workers who care for pregnant women, work atinfant welfare and family planning clinics in all the 30 Local Government Areas were invited for the workshop.

### Data collection

A validated (face validity) questionnaire on the association between pre-eclampsia and cardiovascular risk was distributed to consenting health workers who attended the workshop. The questionnaire was adapted from the questionnaire used at the Beth Israel Deaconess Medical Centre, United States of America [7]. The cadre of health workers and place of practice was included in the questionnaire. The information collected includes socio-demographic characteristics, questions assessing knowledge of pre-eclampsia, future risk of developing cardiovascular complications and counseling on modifiable risk factors.

### Data analysis

The data obtained was entered and analysed with SPSS version 16. Simple descriptive and inferential statistics were done. Knowledge score was computed with each item assigned ‘+1’ for correct knowledge and ‘0’ for incorrect knowledge. The knowledge score ranged from 0 to 7 with mean (SD) 3.95 ± 1.57 and was graded as good (if respondent scored ≥4 points), and not good (if score was <4 points).

Multivariate analysis using binary logistic regression was used to evaluate socio-demographic variables and other variables that are independently associated with good knowledge of future cardiovascular risks. Odds ratios (ORs) and 95 % confidence intervals (CIs) were presented and used as measures of the strength of association. Tests were considered significant for a *p* value < 0.05.

### Ethical consideration

Permission to conduct the study was sought from Osun State Ministry of Health and Osun State Local Government Service Commission. Verbal consent was obtained from every health worker approached to participate in the study. Permission to conduct the study was granted by the Research and Ethics Committee of the Department of Obstetrics & Gynaecology, LAUTECH Teaching hospital, Osogbo.

## Results

### Socio-demographic characteristics

One hundred and forty-six out of 150 health workers approached participated in the study. They were 88 (60.3 %) medical doctors, 19 (13.0 %) nurses/midwives and 39 (26.7 %) community health workers. Mean age of the participants was 35.6 ± 9.1 years (range 22–68 years). The majority of the respondents 67 (45.9 %) were between 18 and 35 years. The median year of practice was 7 years, ranging from 1–40 years. There were 77 (52.7 %) female and 69 (47.3) male respondents, (Table 1).

**Table 1:** Socio-demographic characterics of respondents

Variables	Frequency (%) *N* = *146*
Age (years)	
18–35	67 (45.9)
36–50	62 (42.5)
>50	17 (11.6)
Sex	
Male	69 (47.3)
Female	77 (52.7)
Cadre	
Medical doctors	88 (60.3)
Nurse/midwives	19 (13.0)
Community health workers	39 (26.7)
Place of practice	
Tertiary health facility	13 (8.9)
Secondary health facility	33 (22.6)
Primary health facility	100 (68.5)
Years of practice	
≤10	89 (61.0)
11–20	38 (26.0)
21–30	14 (9.6)
≥31	5 (3.4)
Proportion of treated patients >50 years	
<10 %	33 (22.6)
11–30 %	59 (40.4)
31–50 %	54 (37.0)

The majority of the health workers practised in Primary Health Care facilities (68.5 %) and were in their first decade of practice (61.0 %), (Table 1). Sixty (68.2 %) medical doctors were male, while 18 (94.7 %) nurses/midwives and 31 (79.5 %) community health workers were female (Table 2).

**Table 2:** Association between sex, knowledge of cardiovascular risk and cadre of respondents

Cadre	Sex		Test statistic
	Male (%)	Female (%)	
Medical doctor	60 (68.2)	28 (31.8)	χ^2^ = 42.475; p = 0.001*
Nurse/midwives	1 (5.3)	18 (94.7)	
Community health workers	8 (20.5)	31 (79.5)	
	Knowledge		
	Good	Poor	
Medical doctor	69 (78.4)	19 (21.6)	χ^2^ = 8.951; df = 2; p = 0.011**
Nurse/midwives	11 (57.9)	8 (42.1)	
Community health workers	21 (53.8)	18 (46.2)	

* Fisher exact test

** Pearson Chi-square

### Knowledge of respondents

Medical doctors had better knowledge compared to nurses/midwives and community health workers (78.4 vs. 57.9 vs. 53.8 %; p < 0.05). The majority had good knowledge of future risk of hypertension 127 (87.0 %), ischaemic heart disease 93 (63.7 %), stroke 101 (69.2 %) and kidney disease 107 (73.3 %). Also, the majority 93 (63.7 %) were not aware that short life expectancy is associated with pre-eclampsia, while only 38 (26.0) asked about pre-eclampsia on routine clerking and 67 (45.7 %) counselled on cardiovascular risk, (Table 3).

**Table 3:** Knowledge of health workers on pre-eclampsia and cardiovascular risk

Variables	Yes N (%)	No N (%)	Unsure N (%)
Pre-eclampsia predisposes to increased cancer	11 (7.5)	79 (54.1)	56 (38.4)
Pre-eclampsia predisposes to hypertension later in life	127 (87.0)	7 (4.8)	12 (8.2)
Pre-eclampsia predisposes to ischaemic heart disease	93 (63.7)	15 (10.3)	38 (26.0)
Pre-eclampsia predisposes to stroke	101 (69.2)	17 (11.6)	28 (19.2)
Pre-eclampsia predisposes to kidney disease	107 (73.3)	11 (7.5)	28 (19.2)
Pre-eclampsia predisposes to liver disease	59 (40.4)	19 (13.0)	68 (46.6)
Pre-eclampsia predisposes to shorter life expectancy	51 (34.9)	93 (63.7)	2 (1.4)
Awareness of guidelines in treatment and prevention of hypertension	24 (16.4)	122 (83.6)	
Asking about pre-eclampsia in routing clerking of non-pregnant women	38 (26.0)	108 (74.0)	
Routine counseling on cardiovascular risk in women risk of pre-eclampsia	67 (45.7)	79 (54.3)	
Counseling on general terms of cardiovascular risk	12 (8.2)	134 (91.8)	
Counsel on specific terms such as exercise, stop smoking, salt intake	41 (28.1)	105 (71.9)	

### Factors related to health workers’ knowledge

Majority of the health workers 101 (69.2 %), had good knowledge of the future development of cardiovascular risks by women with pre-eclampsia. Factors determining good knowledge of future cardiovascular risk include professional cadre, sex and research interest in pre-eclampsia Additional file 1.

After adjusting for these factors, only professional cadre remained significant factor of good knowledge, Table 4.

**Table 4:** Binary logistic regression on factors associated with good knowledge of cardiovascular risk

Variable	Crude or (95 % CI)	Adjusted or (95 % CI)
Health worker cadre		
Community health worker	1	1
Nurse/midwives	1.19 (0.40–3.57)	1.16 (0.38–3.58)
Medical doctors	3.13 (1.39–6.99)**	3.14 (1.25–7.88)*
Sex		
Female	1	1
Male	2.00 (0.97–4.13)	1.11 (0.46–2.68)
Research interest in pre-eclampsia		
No	1	1
Yes	1.52 (0.73–3.13)	1.72 (0.80–3.72)

** p < 0.001

* p < 0.05

## Discussion

This study assessed the knowledge of health workers about the association of pre-eclampsia with future cardiovascular disease and their possible offering of any risk-reduction counseling to women with pre-eclampsia. We found that knowledge was high in some specific areas across all professional cadres with medical doctors having better knowledge than other health workers. In our study, there were more females than males respondents, which was similar to the German study but different from the study done in United States of America [13, 14]. Also, most of our participants were in their first decade of practice compared to second decade of practice in the German study [12]. The proportion of women these health workers were attending to aged 51 and above was between 10 and 30 %, similar to the findings in the developed world suggestive of the same exposure to same age group of clients our health workers attend to in the community they are practising [13, 14]. The respondents’ knowledge on risk of future hypertension, ischaemic heart disease, stroke and kidney disease were quite high but lower than the findings in the developed settings. The study showed very low level of awareness on short life expectancy associated with pre-eclampsia which was in contrast to European findings [13, 14]. Below half of the health workers routinely inquired about previous history of pre-eclampsia, while few counselled on risk reduction in women that were at risk of future cardiovascular complications implying that high awareness of the risk has not translated to action taking to reduce the long term morbidity and mortality associated with pre-eclampsia in support of other findings [13, 14]. Many developed countries have guidelines on management of hypertension and pre-eclampsia. Nigeria has a national guideline pattern along the European guideline but that of pre-eclampsia is institution-based. Only 16.4 % were aware of any guideline which was significantly lower than other studies, this might be due to lower cadre of health workers (Primary Health Care workers) involved in this study. Knowledge score showed overall to be quite high, with medical doctors having a significantly better score than other health workers. This is expected as medical doctors’ training is more intense and thorough, thus are better equipped to give better counseling on risk reduction. There was a significant knowledge gap among the health professionals which must be bridged. The lower cadre health workers should be focused since they are closer to the community than the medical doctors, especially in resource constrained countries like Nigeria where majority of the population reside in rural areas. Continuous medical education will enable health workers to gain enough knowledge to counsel these women with pre-eclampsia on risk reduction life-styles. Pre-eclampsia/eclampsia remain a major cause of maternal morbidity and mortality in our country, hence, the health workers should take obstetric history and make appropriate referral for counseling and long term monitoring to reduce the mortality from cardiovascular complications in Nigerian women with pre-eclampsia.

This study is one of the few studies conducted on the health workers’ knowledge of future cardiovascular risk among women with pre-eclampsia in Nigeria. We were unable to identify other studies done in this area in Nigeria. However, since only health workers present at the workshop were approached to participate, it might be difficult to generalize the findings as the respondents may not represent the overall population of health workers in Osun State and beyond.

## Conclusion

Knowledge of cardiovascular risk factors was lower among the nurses/midwives and community health workers. Risk reduction counseling was quite low across all the health workers. There is need for continuous medical education and possible review of the training curriculum of the lower cadres of health workers.

## Additional file

10.1186/s13104-015-1553-6 Health workers knowledge.
