# Supplementary material for: Health workers’ knowledge on future vascular disease risk in women with pre-eclampsia in south western Nigeria
Source: BMC Res Notes. 2015 Oct 16;8:576. doi: 10.1186/s13104-015-1553-6 (PMC4608265; doi:10.1186/s13104-015-1553-6)
Supplement: Supplementary file 1 — 10.1186/s13104-015-1553-6 Health workers knowledge. [file 13104_2015_1553_MOESM1_ESM.doc]

HEALTH WORKERS KNOWLEDGE

Age………………………………………….

Place of Practice 1. PHC 2. State Hospital 3. Comprehensive Health centre 4. LAUTECH

(1) Please indicate your cadre

a. Nursewidewife b. Community health workers c.Doctors

e. General medicine

(2) Please indicate your years of experience as health workers………………………………………………….

(3) Please indicate your gender. a. Male b. Female

(4) What is the age range of the patients that you most commonly encounter

in your practice? a. 18–35 years b. 36–50 years c. >51 years

(5) What is the approximate percentage of women in your practice who are

>50 years of age? a. <10% b. 11–30% c. 31–50%

(6) Do you or a first degree relative have a history of preeclampsia?

a. Yes b. No

(7) Do you have a research interest in preeclampsia?

a. Yes b. No

(8) Do women with preeclampsia have an increased risk of developing these

Diseases later in life?

a. Cancer 1.Yes, 2.No, 3.Unsure

b. Hypertension 1.Yes, 2.No, 3.Unsure

c. Ischemic heart disease 1.Yes, 2.No, 3.Unsure

d. Stroke 1.Yes, 2.No, 3.Unsure

e.Kidney disease 1.Yes, 2.No, 3.Unsure

f. Liver disease 1.Yes, 2.No, 3.Unsure

(9) Do women with a history of preeclampsia have a shorter life expectancy? a. Yes b. No

(10) Are you aware of the Guidelines of Treatment of Hypertension in Prevention

and Management of Ischemic Heart Disease by the American Heart Association? a. Yes b. No

(11) Do the AHA guidelines include history of preeclampsia as a risk factor

for Heart disease? a. Yes b. No c. Don’t know

(12) Does ACOG have a statement about strategies for prevention of cardiovascular

disease in women with preeclampsia? a. Yes b. No c. Don’t know

(13) Do you routinely counsel your patients about the importance of pap smears? a. Yes b. No

(14) Do you routinely counsel patients >51 years old about reducing their cardiovascular

risk? a. Yes b. No

(15) When you take a history from a non pregnant patient, do you routinely

inquire about a history of preeclampsia? (If no, skip to bottom and select

“done” to submit survey.) a. Yes b. No

(16) Do you routinely inquire about the severity of preeclampsia?

a. Yes b. No

(17) Do you routinely inquire about when preeclampsia developed in the

pregnancy? a. Yes b. No

(18) If a woman has a history of preeclampsia, do you counsel her about her

future risk of cardiovascular disease?

a. Yes b. No

(19) If your answer to the above question is “Yes,” what kind of counseling do

you provide?

a. General terms

b. Give specific numbers and indicate her actual risk

c. Give specific information on ways to lower her risk of cardiovascular disease such as diet, exercise, cholesterol levels, blood pressures, smoking cessation, and weight loss
